# Conversion surgery for hepatocellular carcinoma after tyrosine kinase inhibitor treatment

**DOI:** 10.1002/jgh3.12735

**Published:** 2022-04-21

**Authors:** Shun Kaneko, Kaoru Tsuchiya, Yutaka Yasui, Yuki Tanaka, Kento Inada, Shun Ishido, Sakura Kirino, Koji Yamashita, Yuka Hayakawa, Tsubasa Nobusawa, Hiroaki Matsumoto, Tatsuya Kakegawa, Mayu Higuchi, Kenta Takaura, Shohei Tanaka, Chiaki Maeyashiki, Nobuharu Tamaki, Yuka Takahashi, Hiroyuki Nakanishi, Takumi Irie, Shun‐Ichi Ariizumi, Masayuki Kurosaki, Namiki Izumi

**Affiliations:** ^1^ Department of Gastroenterology and Hepatology Musashino Red Cross Hospital Tokyo Japan; ^2^ Department of Surgery Musashino Red Cross Hospital Tokyo Japan; ^3^ Department of Surgery, Institute of Gastroenterology Tokyo Women's Medical University Tokyo Japan

**Keywords:** albumin–bilirubin score, conversion surgery, hepatocellular carcinoma, lenvatinib, sorafenib, tyrosine kinase inhibitor

## Abstract

**Background and Aim:**

Conversion surgery (CS), which aims to cure after systematic therapy, is only scarcely reported in the field of hepatocellular carcinoma (HCC). However, advancements in systemic therapy for HCC are expected to increase the candidates eligible for CS because of the higher response rate. The aim of this study was to clarify the characteristics of patients who underwent CS after tyrosine kinase inhibitor (TKI) therapy.

**Methods:**

In all, 364 patients who were treated with first‐line sorafenib (SOR; *n* = 292) and lenvatinib (LEN; *n* = 72) from July 2009 to October 2020 were retrospectively enrolled. The endpoint of this analysis was overall survival (OS), and factors associated with CS are revealed.

**Results:**

Six patients underwent CS after TKI therapy, and of these four (1.4%) and two (2.7%) patients received SOR and LEN, respectively. At baseline, patients who underwent CS were significantly younger (median 52 [range, 46–83] years of age, *P* = 0.019), and their etiology included viral hepatitis, especially hepatitis B virus (HBV) (*P* = 0.049). Improvements or maintenance of preoperative modified albumin–bilirubin grade from baseline were observed in five (83.3%) patients, and partial radiologic response was observed in four (66.7%) patients. The median OS and 3‐year survival rate of patients treated with CS were “not reached” and 80.0%, respectively.

**Conclusion:**

The patients who underwent CS after TKI therapy for HCC experienced long survival, were relatively young, and exhibited radiologic response to TKIs, and their liver function was either maintained or improved. Therefore, CS may lead to a better prognosis in patients with advanced HCC.

## Graphical abstract

The patients who underwent conversion surgery (CS) after tyrosine kinase inhibitor (TKI) therapy for hepatocellular carcinoma experienced long survival. The patients who underwent CS were relatively young, and exhibited radiological response to TKIs, and their liver function was either maintained or improved.

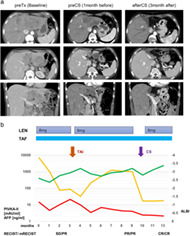

## Introduction

The development of drug therapies for advanced hepatocellular carcinoma (HCC) has made remarkable progress since sorafenib (SOR)^1^ was approved for the treatment of unresectable HCC. Recently, first‐line therapy for the systemic treatment of advanced HCC has dramatically shifted. In 2018, the REFLECT trial showed the non‐inferiority of lenvatinib (LEN) over SOR as first‐line therapy for patients with unresectable HCC, and the overall response rate was higher for LEN than for SOR (41 *vs* 12% for mRECIST criteria and 19 *vs* 7% for RECIST 1.1).^2^ Ultimately, achieving complete response (CR) has been shown to contribute most to improving prognosis.^3^, ^4^, ^5^ However, CR is often difficult to achieve with drug therapy alone. In addition to advances in systematic therapy, some studies have reported longer survival times after surgery/transarterial chemoembolization (TACE) after chemotherapy to achieve higher therapeutic effects.^6^, ^7^, ^8^, ^9^, ^10^, ^11^, ^12^, ^13^

Among patients with advanced HCC treated with SOR, there have been several long‐term survivors after conversion surgery (CS) and SOR treatment.^6^, ^7^ Only a small proportion of patients with unresectable HCC could achieve downstaging and undergo resection following SOR treatment because of the relatively low objective response rate (ORR; <10%).^1^, ^8^ Then, more several case reports have been published about patients with HCC undergoing CS after multidisciplinary treatment including LEN.^9^, ^10^, ^11^ LEN–TACE sequential therapy rather than TACE monotherapy has been proposed for patients with HCC classified as Barcelona Clinic Liver Cancer (BCLC) stage B with a high tumor burden.^12^, ^13^

It has been reported that a higher response rate can lead to better outcomes after liver resection in patients treated with conversion chemotherapy for initially unresectable colorectal liver metastases.^14^ From this point of view, LEN has become the preferred targeted therapy to achieve CS because of its higher tumor necrosis effect, as evaluated by mRECIST and RECIST 1.1.^15^, ^16^ In one LEN study, all patients had a tumor with a main portal vein tumor thrombus (PVTT; Vp4) or >50% liver occupation. Patients with tumors and Vp4 had a response rate of 20.0%, whereas those with tumors with >50% liver occupation had a response rate of 29.3% according to mRECIST.^17^ Recently, data of patients with advanced HCC who underwent conversion surgery after LEN therapy have accumulated. The conversion rate for curative resection was 8.4% (9/107). Curative resection showed an independent association with better disease‐specific survival (hazard ratio [HR] 0.04, 95% confidence interval 0.01–0.30; *P* = 0.002). Additional treatments other than curative resection showed fewer or marginal survival benefits.^18^

As described above, the number of patients who undergo CS after systematic therapy will increase in the future as the response rate increases as a result of advancements in systematic therapy for HCC. The aims of this study are to clarify the characteristics of patients who underwent CS after first‐line TKI (SOR and LEN) treatment and to propose this strategy for advanced HCC.

## Methods

### Patients

In all, 364 patients treated with first‐line SOR or LEN at the Musashino Red Cross Hospital from July 2009 to October 2020 were retrospectively enrolled. The diagnosis of HCC was based on pathological or radiologic findings, such as typical arterial enhancement of the tumor followed by a washout pattern in the portal venous phase or the equilibrium phase on dynamic computed tomography (CT) or magnetic resonance imaging, in accordance with practice guidelines.^19^ The extent of PVTT was classified as follows: Vp0, no PVTT; Vp1, segmental PV invasion; Vp2, right anterior or posterior PV; Vp3, right or left PV; and Vp4, main trunk and/or contralateral PV branch to the primarily involved lobe.^20^, ^21^, ^22^

Inclusion criteria for TKI treatment were as follows: metastatic or locally advanced HCC that was unresectable or refractory to TACE; primarily BCLC stage B or C^23^; and an Eastern Cooperative Oncology Group performance status of 0 to 1.^24^

Child–Pugh (C–P) classification as well as the albumin–bilirubin (ALBI) and modified ALBI (mALBI) grades were used to assess liver function. The ALBI score was calculated on the basis of serum albumin and total bilirubin values as previously reported.^25^ The ALBI grade was defined as follows: grade 1, ≤−2.60; grade 2, >−2.60 to ≤−1.39; and grade 3, >−1.39. Furthermore, grade 2 ALBI was divided into two subgrades (2a and 2b) using a cutoff value (ALBI score −2.270), and such ALBI grades were defined as mALBI grades.^26^ To evaluate the radiologic response, we evaluated the response according to RECIST 1.1 and mRECIST, as previously reported.^3^, ^4^, ^15^, ^16^

Written informed consent was obtained from all study participants. This study was approved by the ethics committee of the Musashino Red Cross Hospital. The investigation was conducted in accordance with the Declaration of Helsinki.

### Tyrosine kinase inhibitor therapy

SOR was orally administered at a dose of 800 mg/day. A reduced initial dose was allowed for patients with advanced age (≥80 years) and low body weight (<50 kg). LEN was orally administered at a dose of 8 mg/day to patients weighing <60 kg and 12 mg/day to those weighing ≥60 kg. SOR and LEN were reduced or interrupted if a patient developed any unacceptable grade 2 or any grade 3 drug‐related adverse events (AEs). If a patient developed AEs, dose reduction or temporary interruption was maintained until the symptoms resolved to less than grade 2. AEs were assessed according to the National Cancer Institute Common Terminology Criteria for Adverse Events, version 5.0.^27^

### Study endpoint

The endpoint of this analysis was OS, which was the time from TKI treatment initiation until death by any cause or the last follow‐up. Baseline factors between patients who received TKI with or without CS were compared. The factors analyzed for significance included age, sex, chronic liver disease etiology, serum data (albumin, bilirubin, and alpha‐fetoprotein [AFP]), C–P score, ALBI score, HCC condition (portal vein invasion, extrahepatic metastasis [EM], and BCLC stage), first‐line TKI (SOR or LEN), and TKI therapy duration.

### Statistical analyses

Chi‐squared and Fisher's exact tests were used to compare categorical data. Student's *t*‐test or Mann–Whitney *U*‐test was used to analyze the distribution of continuous variables. Cumulative survival rates of patients were determined using the Kaplan–Meier method. A *P*‐value <0.05 was considered statistically significant. GraphPad Prism software (GraphPad Software, San Diego, CA, USA) was used to analyze statistical significance.

## Results

### Case report

**Figure 1:**
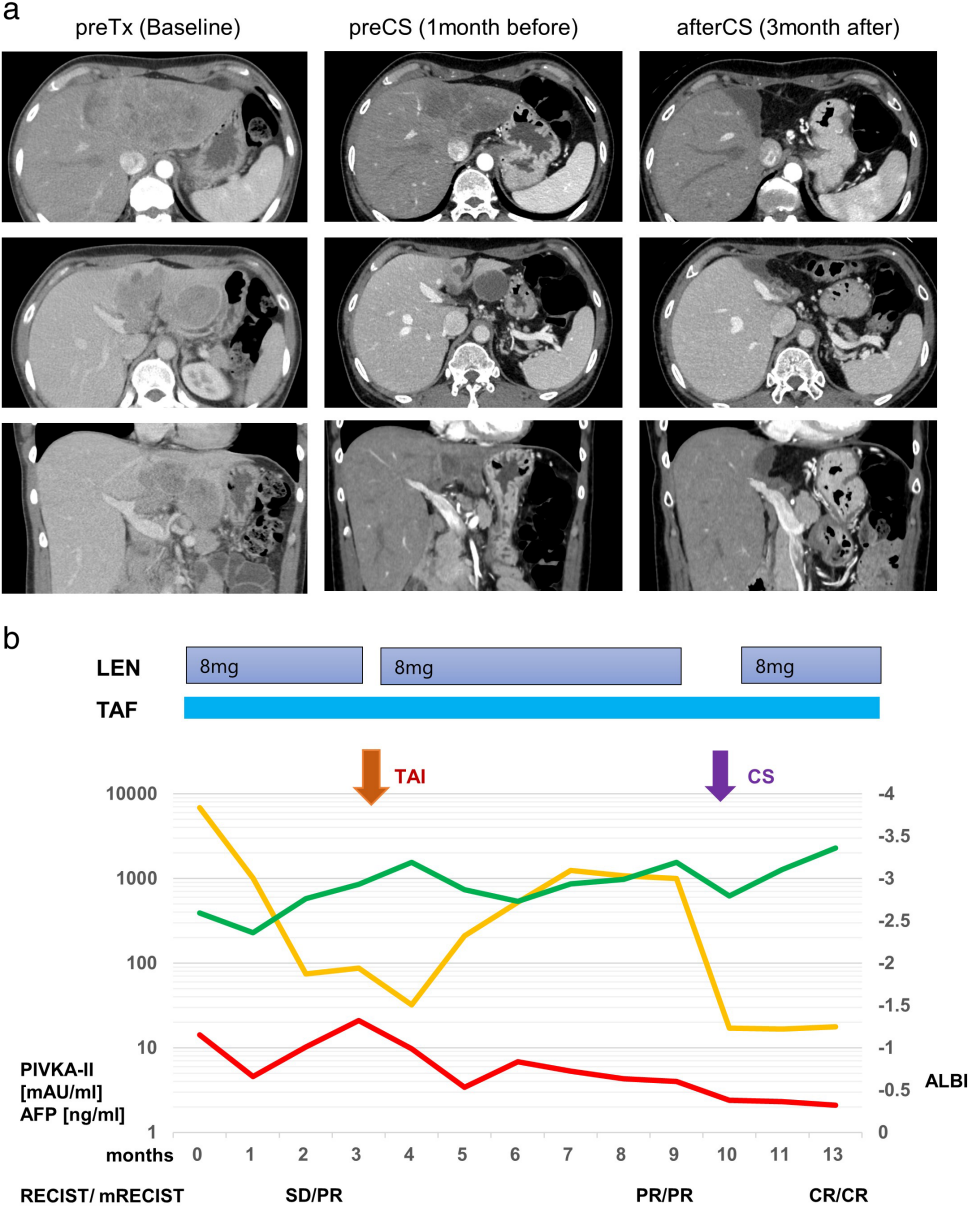
Clinical course of a 48‐year‐old female patient with hepatocellular carcinoma (case 1). (a) Abdominal dynamic computed tomography at diagnosis, at 1 month before conversion surgery (CS), and at 3 months after CS. (b) Changes in tumor markers (prothrombin induced by vitamin K absence‐II [PIVKA‐II] and alpha‐fetoprotein [AFP]) and albumin–bilirubin (ALBI) score. CR, complete response; LEN, lenvatinib; mRECIST, modified Response Evaluation Criteria in Solid Tumors; PR, partial response; RECIST, Response Evaluation Criteria in Solid Tumors; SD, stable disease; TAF, tenofovir alafenamide fumarate; TAI, transcatheter arterial infusion.

A 48‐year‐old female was referred to the Musashino Red Cross Hospital for back pain, and a liver tumor was discovered by ultrasonography and CT scan. She had no significant medical history. Abdominal dynamic CT scan revealed a tumor 13.4 cm in size in the left lobe of the liver with invasion of the left PV (Vp3) (Fig. 1a). No extrahepatic lesions were observed. Her serum AFP and prothrombin induced by vitamin K absence‐II (PIVKA‐II) levels had increased to 14.5 ng/mL and 7547.8 mAU/mL, respectively. Poorly differentiated HCC was pathologically observed by liver biopsy. Based on these findings, she was diagnosed with BCLC stage C advanced HCC.

With respect to background liver disease, she was positive for hepatitis B surface antigen (HBsAg) and HBV DNA, with titers of 1.92 IU/mL and 4.6 log IU/mL, respectively. Her alanine aminotransferase (ALT) level was 20 U/L. She was diagnosed with chronic hepatitis B (Genotype C, HBcrAg 6.3 log IU/mL, HBeAg‐positive, and HBV DNA 4.6 IU/mL) and immediately treated with tenofovir alafenamide fumarate (TAF). Her liver function showed a C–P score of A (5 points) and an ALBI score of −2.594. LEN was administrated at 8 mg/day. No AEs occurred, and therefore she could be treated without LEN reduction. Two months after the initial treatment, dynamic CT revealed no change in tumor size but it did reveal decreased intratumor vascularity, which was determined to be stable according to RECIST 1.1 and partial response by mRECIST (Fig. 1b). However, a decrease in the size of PVTT was not shown. Based on these results, transcatheter arterial infusion (TAI) with cisplatin was initiated to achieve further therapeutic effects. LEN was stopped 2 days before TAI and restarted 1 week later.

**Figure 2:**
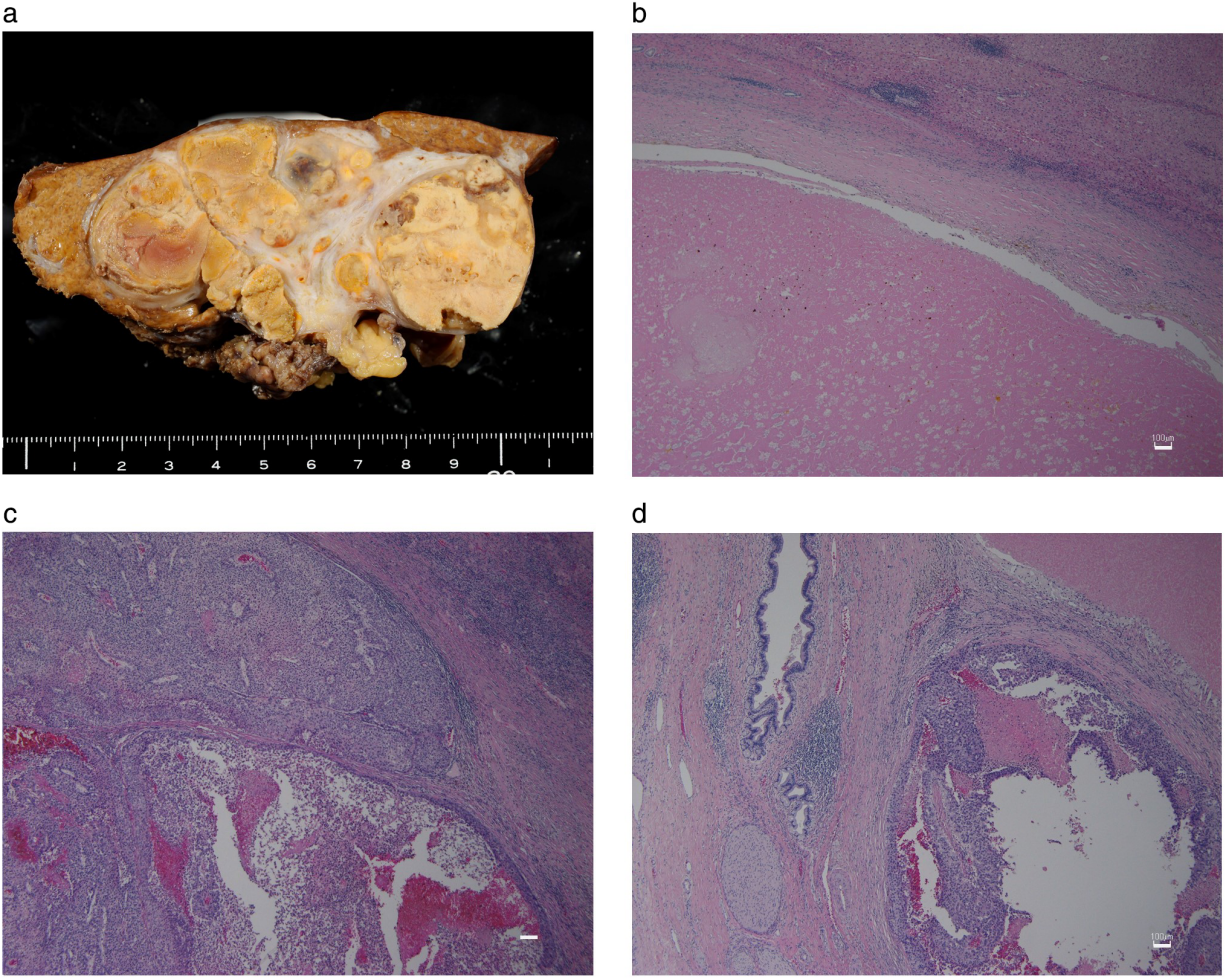
Pathological findings of hepatocellular carcinoma (HCC) from conversion therapy (case 1). (a) Macroscopic findings in a surgical specimen. (b–d) Microscopic findings in a surgical specimen, Scale bars: 100 μm. (b) Most of the tumor was occupied by a necrotic lesion. Some viable HCC cells (poorly differentiated HCC) remained in the intrahepatic tumor (c) and portal vein tumor thrombus (d).

After 8 months of LEN treatment, both the AFP and PIVKA‐II levels had significantly declined to 4.5 ng/mL and 1072.1 mAU/mL, respectively, and dynamic CT revealed PR according to RECIST 1.1 and mRECIST (Fig. 1a,b). Based on this clinical course, CS was considered to achieve CR as a further therapeutic effect. Enlargement of the left liver, caudate lobectomy, and portal vein tumor resection were performed at the Tokyo Women's Medical University approximately 9 months after the initiation of LEN (Fig. 2) (depending on the patients' wishes). LEN was stopped 2 days before CS and restarted 4 weeks later. A microscopic histopathological examination revealed that most of the tumor was occupied by a necrotic lesion. However, some viable HCC cells (poorly differentiated HCC) remained in the intrahepatic tumor and PVTT (Fig. 2).

She then continued LEN treatment, experienced no recurrence at 5 months after LEN administration, and maintained CR status.

### Retrospective study

#### Patient characteristics

Between July 2009 and October 2020, 364 patients began TKI therapy, and of these, 292 and 72 patients received SOR and LEN, respectively, as first‐line TKI therapy. The patient characteristics are presented in Table 1. The median age was 74 (range, 26–98) years. The number of patients with C–P grade A was 320 (87.9%), while the number of those with grade B was 44 (12.1%). The median ALBI score was −2.278. Overall, 165 (45.3%) and 192 (52.7%) patients had BCLC stage B (intermediate) and C (advanced), respectively. Moreover, 134 (36.8%) patients had EM. Regarding tumor PVTT, 234 (64.3%), 55 (15.1%), and 72 (20.6%) patients had Vp0, 1/2, and 3/4, respectively. The median TKI treatment duration was 115 days.

**Table 1:** Clinical characteristics of advance hepatocellular carcinoma patients with or without conversion surgery at baseline

	All	Conversion surgery–	Conversion surgery+	*P*‐value
*n*	364	358	6	
Age (years), median (range)	74 (26–98)	74 (26–98)	52 (46–83)	0.0197*
Sex: male/female (%)	285 (78.3%)/79 (21.7%)	281 (78.5%)/77 (21.5%)	4 (66.7%)/2 (33.3%)	0.614
Etiology of chronic liver disease				
HBV	50 (13.7%)	47 (13.1%)	3 (50.0%)	0.0495*
HCV	181 (49.7%)	180 (50.3%)	1 (16.7%)	
Alcohol	41 (11.3%)	41 (11.5%)	0 (0%)	
Others	92 (25.2%)	90 (25.1%)	2 (33.3%)	
Child–Pugh class				
A	320 (87.9%)	314 (87.7%)	6 (100%)	1
B	44 (12.1%)	44 (12.3%)	0 (0%)	
ALBI score	−2.278 (−3.381 to –1.062)	−2.251 (−3.381 to –1.062)	−2.478 (−2.956 to –2.189)	0.0669
mALBI grade 1/2a/2b/3	89/90/168/17	87/87/167/17	2/3/1/0	
BCLC				1
B (intermediate stage)	165 (45.3%)	163 (45.5%)	2 (33.3%)	
C (advanced stage)	192 (52.7%)	189 (52.8%)	3 (50.0%)	
Extrahepatic spread				0.421
Yes	134 (36.8%)	133 (37.1%)	1 (16.7%)	
No	230 (63.2%)	225 (62.9%)	5 (83.3%)	
PVTT Vp 0/1, 2/3, 4	234/55/75	231/53/74	3/2/1	0.387
Median AFP concentration at baseline	120 (1.6 to 143 979)	124.95 (1.6 to 143 979)	45.85 (7.4 to 5410)	0.826
Additional therapy	RT 53/HAIC 43	RT 52/HAIC 41	RT 1/HAIC 2	
Median TKI therapy duration (days)	115 (10–2739)	110 (10–2739)	278.5 (56–1513)	0.099
First TKI SOR/LEN	292 (80.2%)/72 (19.8%)	288 (80.4%)/70 (19.6%)	4 (66.7%)/2 (33.3%)	0.339

AFP, alpha fetoprotein; ALBI, albumin–bilirubin; BCLC, Barcelona Clinic Liver Cancer; HAIC, hepatic arterial infusion chemotherapy; HBV, hepatitis B virus; HCV, hepatitis C virus; LEN, lenvatinib; mALBI, modified ALBI; PVTT, portal vein tumor thrombosis; RT, radiotherapy; SOR, sorafenib; TKI, tyrosine kinase inhibitor.

* *P*‐value < 0.05.

#### Baseline characteristics of patients who underwent CS

Six patients underwent CS after TKI therapy, and of these, four (1.4%, 4/292) and two (2.7%, 2/72) patients received SOR and LEN, respectively, as first‐line TKI therapy (Table 1). The median age was 52 (range, 46–83) years, and the etiology of chronic liver disease included HBV (*n* = 3, 50.0%) and HCV (*n* = 1, 16.7%). The median ALBI score was −2.478 (range, −2.956 to –2.189). The patients who underwent CS were significantly younger (*P* = 0.019) and their etiology included viral hepatitis, especially HBV (*P* = 0.049), compared with patients who did not undergo CS. The median ALBI score tended to be lower in CS patients (*P* = 0.06, marginally significant). No significant differences were observed in sex, C–P Class, BCLC stage, EM, the extent of PVTT, median AFP concentration, median TKI therapy duration, or first‐line TKI regimen.

#### Overall survival of patients treated with CS

**Figure 3:**
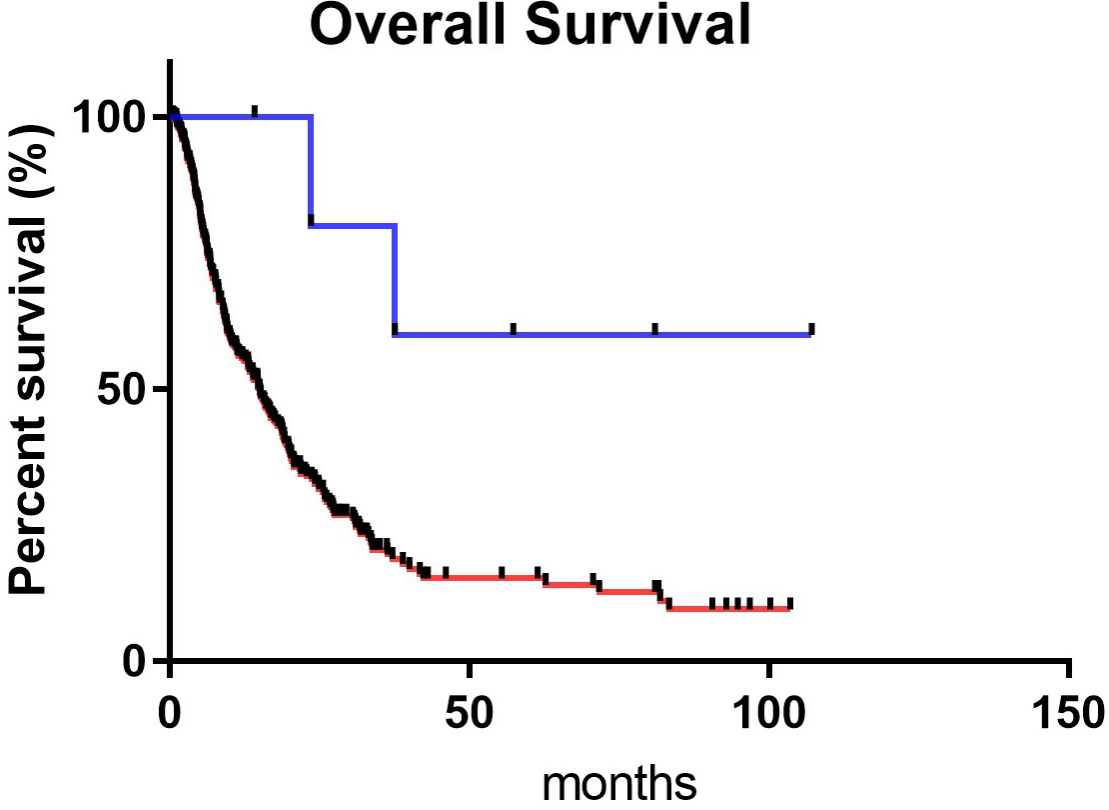
Overall survival of patients who underwent conversion surgery after treatment with tyrosine kinase inhibitors for hepatocellular carcinoma.

Survival curves, according to whether patients underwent CS (*n* = 6) or without CS (*n* = 358), are shown in Figure 3. The median OS of patients treated with or without CS were “not reached” and 14.9 months, respectively. The 3‐year survival of those were 80.0 and 20.5%, respectively.

#### Characteristics of patients who underwent CS

Detailed characteristics of patients who underwent CS are presented in Table 2.

**Table 2:** Clinical course and characteristics of patients who received conversion surgery after tyrosine kinase inhibitor (TKI)

2a: At baseline																	
Case no.	First TKI	Age	Sex	Duration of TKI (days)	Additional Tx	Etiology	AntiviralTx	Max. tumor size (mm)	Tumor number	Position	Vascular invation	Extraheptic metastasis	AFP (ng/mL)	PIVKA (mAU/mL)	mALBI Grade	Child–Pugh	
1	LEN	48	F	388	TAI	HBV	TAF	134	1	Left lobe	Vp3	None	14.2	6818.6	2a	5	A
2	LEN	49	M	56	HAIC	NBNC		87	5	Right + left	Vp2	None	1969	2870	2b	6	A
3	SOR	46	M	1513	HAIC/TACE	HBV	LAM	42	Multi	Right lobe	0	Bone	7.4	186	1	5	A
4	SOR	83	M	414		NBNC		130	Multi	Right + left	0	None	5410	847 000	2a	5	A
5	SOR	69	F	169	S‐1	HCV	none	16	2	Right lobe	0	None	69.6	45	1	5	A
6	SOR	55	M	98	RT	HBV	ETV → TAF	100	2	Right + left	Vp2Vv3	None	22.1		2a	5	A

2b: At conversion surgery												
Case no.	Survival	Survival month	Before CS						Pathology	Post CS		
			AFP (ng/mL)	PIVKA (mAU/mL)	mALBI Grade	Child–Pugh		RECIST1.1		RECIST1.1	Recurrence time after surgery (months)	Tx at recurrence or maintenance
1	Alive	14.10	4.3	1072.1	1	5	A	PR	por	CR	ND	LEN
2	Dead	23.50	18.3	44.9	2a	5	A	PR	por	CR	1.43	HAIC
3	Alive	80.93	783	1530	1	5	A	SD	mod > well	CR	5.60	TACE
4	Alive	57.27	91.6	24 000	2a	5	A	PR	por > mod	CR	9.10	RFA
5	Dead	37.53	1720	88	1	5	A	PD	mod > well Vp1	CR	6.40	RFA
6	Alive	107.13	4.2	57	2a	6	A	PR	mod > well Vp1	PR (residual HCC in IVC)	4.57	UFT → TACE

AFP, alpha fetoprotein; ALBI, albumin–bilirubin; CR, complete response; ETV, entecavir; HAIC, hepatic arterial infusion chemotherapy; HBV, hepatitis B virus; HCV, hepatitis C virus; LAM, lamivudine; LEN, lenvatinib; mALBI, modified ALBI; PD, progressive disease; PIVKA‐II, protein induced by vitamin K absence or antagonist‐II; PR, partial response; RECIST, Response Evaluation Criteria in Solid Tumors; RFA, radiofrequency ablation; RT, radiotherapy; SD, stable disease; SOR, sorafenib; TACE, transcatheter arterial chemoembolization; TAF, tenofovir alafenamide fumarate; TAI, transcatheter arterial infusion; Tx, therapy.

At the time of TKI introduction, the maximum tumor diameter was 93.5 (range, 16–134) mm, the median number of tumors was 5 (range, 1–10 or more), and five cases (83.3%) were out of the “up‐to‐seven” criteria. The median period of TKI administration was 278.5 (range, 56–1513) days. Improvements in and maintenance of the preoperative mALBI grade were observed in two and three cases, respectively, compared with those at the time of introduction of TKI therapy. Though the ORR of patients received TKI without CS was 24.0%, radiologic partial response was observed in four (66.7%) cases in patients who underwent CS. One case was BCLC stage A, which was unrefractory to TACE and SOR therapy, and therefore CS was performed. According to the pathological findings, three cases were moderately differentiated and three were poorly differentiated. HCC recurrence was observed in five cases (83.3%) at a median of 5.6 (range, 1.4–9.1) months after surgery.

Based on these results, the patients who underwent CS were relatively young and responded to TKI treatment, and their liver function was either maintained or improved.

## Discussion

This study provides valuable evidence of the benefits of CS after first‐line TKI (SOR and LEN) therapy in terms of a better prognosis in patients with advanced HCC. Based on a comparison of characteristics of patients who underwent CS and those who did not, patients who underwent CS were relatively young and responded well to TKIs, and their liver function was either maintained or improved. Six patients underwent CS after TKI therapy, and of these, four (1.4%, 4/292) and two (2.7%, 2/72) received SOR and LEN, respectively, as a first‐line TKI therapy. Although no significant differences were observed, the proportion of patients who underwent CS was numerically higher in patients who received LEN than SOR. This might reflect differences in the ORR.

In our presented case (Figs 1, 2), the administration of LEN led to CS and a better prognosis. She received continuous LEN therapy and TAI therapy for PVTT as per the rationale below. We previously had reported that ALBI 1/2a patients who had HCC with PVTT had a lower risk of TKI discontinuation due to deterioration of liver function than ALBI 2b/3 patients.^22^ The LEOPARD study to evaluate the efficacy and safety of LEN in combination with HAI using cisplatin had reported high ORR (RECIST/RECISTv1.1: 64.7%/45.7%).^28^ Based on these reports, we considered that TAI enhanced the effect of the LEN therapy. Furthermore, some indications suggested by this case are as follows. The first indication is “effective response to LEN for advanced HCC.” No AEs occurred, and therefore she could be treated without LEN reduction. After 8 months of LEN treatment and TAI, both AFP and PIVKA‐II levels had significantly declined to 4.5 ng/mL and 1072.1 mAU/mL, respectively, and dynamic CT revealed PR according to RECIST 1.1 and mRECIST (Fig. 1a). This may affect the resection range and the safety of surgery. The second indication is “maintenance of background liver function.” She was diagnosed with chronic hepatitis B and was immediately treated with TAF. Fortunately, her liver was not cirrhotic and did not exhibit decreased liver function; therefore, antiviral therapy could be administered in parallel with HCC treatment. The increased ALBI score was considered to be due to the large areas of HCC in the liver, and improvement was seen after successful treatment with LEN. Finally, she underwent CS, which was effective. The evaluation of liver function in advanced HCC is focused on one prognostic factor^25^, ^26^ and is used for decisions on treatment strategy,^22^ especially evaluation by the ALBI grade. The third indication is “timing of CS and adjuvant therapy.” Determination of the timing of surgery is still controversial. Early surgery may be risky and lead to failure, to take advantage of combination therapy, whereas secondary drug resistance and tumor progression may occur as a result of late surgery. In this case, CS was performed at a point where these two conditions, namely effective treatment (PR) and improved liver function resulting from LEN, were met. The half‐life of LEN in the plasma is short at approximately 28–35 h.^29^ Therefore, cessation 2 days prior to surgery seemed to be sufficient before CS. Another point is that LEN might be needed as an adjuvant therapy. Although CS led to a radiologic CR, some viable HCC cells (poorly differentiated HCC) remained in the intrahepatic tumor and PVTT in the resected tissue (Fig. 2). Regarding case 2, the patient was not willing to resume LEN and hepatic arterial infusion chemotherapy after CS, and, finally, disease control became difficult because of intense recurrence. The poorly differentiated histological type may also be considered as a cause of disease intensity.^30^ Although evidence has not yet been established, adjuvant therapy (resumption of TKI) should be considered in such cases.

In this retrospective study, the median OS of patients who received CS was long (Fig. 3), and the baseline characteristics were compared between those who underwent CS and those who did not (Table 1). The patients who underwent CS were significantly younger and their disease etiology included viral hepatitis, especially HBV. The median ALBI score tended to be lower in CS patients. Among the six CS patients, improvements in and maintenance of the preoperative mALBI grade were observed in five (83.3%) patients and partial radiologic response was observed in four (66.7%) patients (Table 2). Based on these results, the tumor shrinkage effect of TKI is necessary, and it is also important to manage background liver function. Specifically, in hepatitis B patients, HBV can be treated by nucleoside and nucleotide analogs even during parallel treatment for HCC.^31^ In addition, it might be possible to manage viral hepatitis earlier, which is highly likely to be followed up.

This study had several limitations. First, this was a case report and retrospective study in which the exclusion of unidentified biases was impossible. The small number of patients in this study precluded the performance of more solid comparison analyses. It would have been more persuasive if we had compared patients who had CS after TKIs with those who had surgery from the beginning. Second, regarding CS, we were unable to determine the best method or timing of the surgery. Recommended treatments were discussed and decided by the tumor board; however, the preference of each patient strongly influenced the final decision. A follow‐up study of a larger cohort is needed for further analysis, including the prognostic factors after CS. A multicenter, single‐arm observational study of LEN followed by surgical resection for initially unresectable HCC is in progress and results are expected. Third, systemic treatment options to be administered to advanced HCC during the observation period have changed significantly over the last decade. Not only can systematic chemotherapy now be used with SOR and LEN, but combination therapy of atezolizumab and bevacizumab (ATZ + BEV) was approved because of its ability to significantly prolong the progression‐free survival and OS of patients with unresectable HCC compared with SOR alone when used as a first‐line therapy.^32^ Recently, some studies have reported patients with initially unresectable HCC who received successful conversion therapy with anti‐PD‐1 antibodies and combined TKI/anti‐PD‐1 antibodies.^33^, ^34^ With the advent of these highly efficacious chemotherapy regimens, the number of patients undergoing CS cases may increase in the future.

In conclusion, CS after TKI treatment is a useful strategy for advanced HCC. When patients start systematic therapy, they should be considered for CS if an antitumor effect and good background liver function are observed. The application of CS may lead to better prognosis in terms of longer survival in patients with advanced HCC.
